# Anesthesia Dolorosa of Trigeminal Nerve, a Rare Complication of Acoustic Neuroma Surgery

**DOI:** 10.1155/2014/496794

**Published:** 2014-09-25

**Authors:** Foad Elahi, Kwo Wei David Ho

**Affiliations:** Center of Pain Medicine, University of Iowa, 200 Hawkins Drive, Iowa City, IA 52242, USA

## Abstract

Anesthesia dolorosa is an uncommon deafferentation pain that can occur after traumatic or surgical injury to the trigeminal nerve. This creates spontaneous pain signals without nociceptive stimuli. Compression of the trigeminal nerve due to acoustic neuromas or other structures near the cerebellopontine angle (CPA) can cause trigeminal neuralgia, but the occurrence of anesthesia dolorosa subsequent to acoustic tumor removal has not been described in the medical literature. We report two cases of acoustic neuroma surgery presented with anesthesia dolorosa along the trigeminal nerve distribution. The patients' pain was managed with multidisciplinary approaches with moderate success.

## 1. Introduction

Improved diagnosis and surgical treatments have lowered the morbidity and mortality of acoustic neuromas over the past few decades. However, there are accumulating evidences showing that the removal of acoustic neuroma is often associated with complications.

Among those complications, facial weakness and headache are the most common [1]. The prevalence of headache after acoustic neuroma surgery is reported to be from 23% to 34% at 3 months [2, 3], from 16% to 29.5% at 1 year after surgery [2, 4, 5], and 9% at 2 years [2].

Anesthesia dolorosa is a chronic pain condition where patients experience numbness in facial areas but at the same time also have constant severe pain. This injury arises from injuries to the first order trigeminal nerve, thus causing second-order neurons on the trigeminal pain pathway to spontaneously fire, producing pain signals without nociceptive stimulus [6]. It occurs among 2% to 4% of patients who have undergone trigeminal rhizotomy [7, 8]. Though anesthesia dolorosa is a well-described complication of trigeminal rhizotomy, it is not usually associated with acoustic neuroma surgery. There is no evidence in the medical literature about occurrence of the anesthesia dolorosa either subsequent to acoustic neuroma due to tumor involvement or subsequent to surgical intervention.

Among complications of acoustic neuroma surgery, the prevalence of symptoms affecting the face or tongue, including numbness or pain, is reported to be 10.7% [9]. However, the prevalence of anesthesia dolorosa after acoustic neuroma surgery has not been documented.

Here we will describe two cases of anesthesia dolorosa following acoustic neuroma surgery. These two cases both presented with chronic deafferentation pain with typical sensory loss (anesthesia dolorosa) in the dermatome pattern of the trigeminal nerve after acoustic neuroma surgery.

## 2. Case 1

The patient is a 63-year-old gentleman who presents to the pain clinic for evaluation of right facial pain. He was diagnosed with right side acoustic neuroma occupying the cerebellopontine angle (CPA). The patient's history is significant for three surgeries with the final outcome of total removal of the acoustic neuroma via CPA approaches by the neurosurgeon. The patient states that he has had pain for about 3 to 4 years and was aggravated after his last surgery. He denied having headaches.

He states that the pain started after the first surgery and it was initially controlled when he was started on gabapentin but has progressively worsened to what it is at this point. The pain is constant, electrical, and sharp in the distribution of the right mid face mostly at maxillary (V2) and partially at mandibular (V3). The pain was not aggravated by chewing or blowing of cold air. He rated his pain score at most times a 10 and occasionally at best about 5 on the numerical pain rating scale (NPR). The pain is on the right side of his face, even though his entire face feels numb.

He states that the right facial pain feels like someone “threw a bolt of lightning at his face.”

The patient recent MRI imaging showed no tumor recurrence.

On physical examination, he had right side sensory loss to light touch and pinprick on V2 and V3 distribution. Due to a complete right side facial palsy he had a right partial blepharoplasty. There was no trigger point on examination. With the diagnosis of anesthesia dolorosa along the right V2 and V3 distribution we provided education about multimodality pain treatment strategies. A combination of antiepileptic and antidepressant antalgic medications including gabapentin 3600 mg daily and amitriptyline 150 mg was initiated. On his follow-up we discussed the possibility of sphenopalatine ganglion block as a diagnostic tool to understand to what degree the autonomic nervous system played a role in his treatment. Sphenopalatine ganglion block on two occasions was performed under fluoroscopy guidance. The sphenopalatine ganglion block did not provide additional pain relief. The patient is well-trained with the assistance of a pain psychologist to perform biofeedback and self-hypnosis. He was also educated about coping skills. On his six-month follow-up, he rated his pain at a 5 (NPR) and he mentioned that he is able to accept his pain at this level and considered this a success.

## 3. Case 2

This patient is a 37-year-old female who presents to the pain clinic with a chief complaint of right side facial pain and numbness. The patient reports that the pain becomes more and more significant during the day until she feels the pain radiating from the right side of the entire face to the upper neck.

The patient has a history of an acoustic neuroma surgery with near total tumor removal. The surgery was performed 10 years ago. She underwent stereotactic radiosurgery for the tumor remnant shortly after surgery. The patient developed right facial palsy immediately after the surgery. She reported that she was having right facial pain and gradually was losing touch feeling on the face area after the stereotactic radiosurgery course was finished. The pain and numbness have persisted ever since. The right face numbness did not improve and the pain intensity did not change during the past nine years.

The patient characterizes her pain as a sharp, constant pain and rated it as an 8 out of 10 on numerical pain rating scale (NPR). The pain is occasionally exacerbated with chewing. Recently there was an MRI image performed that showed a small, stable-sized remnant lesion of the prior tumor along the CPA region. The stable size of the tumor during the past nine years did not warrant surgical intervention or further radiation therapy.

On physical examination, the patient had complete loss of sensation to light touch and pinprick on the right ophthalmic (V1), maxillary (V2), and mandibular (V3) dermatomes. She had a right side blepharoplasty due to right side complete facial palsy. She was under the multimodality treatment strategy that included a combination of antiepileptic antalgic medication, antidepressant antalgic medication, and opioid medication management with occasional rotation along with applying psychological techniques such as coping, biofeedback, and hypnosis. All the prior efforts including sphenopalatine ganglion block, trigeminal nerve blocks, and trigeminal nerve radiofrequency were unsuccessful in her treatment. With the diagnosis of anesthesia dolorosa, a complicated deafferentation centralized pain, we may consider deep brain stimulation in the future.

## 4. Discussion

Here we report two cases of chronic centralized pain in the category of the anesthesia dolorosa after acoustic neuroma surgery. Both patients have had many years of chronic constant pain on one side of the face as well as numbness.

Anesthesia dolorosa is characterized by persistent neuropathic pain with numbness along the territory of the respected nerve damage. The causes for nerve damage and the traumatic event can be mechanical, thermal, chemically induced, or from radiation.

Acoustic neuroma (vestibular schwannoma) surgery, depending on the surgical approach and tumor characteristics, has various complications. Surgical complications of acoustic neuroma with different surgical approaches have been systemically reviewed [10]. The middle cranial fossa approach, retrosigmoid (CPA) approach, and translabyrinthine approach were the three approaches evaluated. Postoperative headache, facial nerve dysfunction, hearing loss, and cerebrospinal fluid leak are the most common complications. For postoperative headache, it was significantly more likely after the retrosigmoid approach than after the translabyrinthine approach, but neither differed significantly from the middle cranial fossa approach. For facial nerve complications, the retrosigmoid approach was associated with significantly less dysfunction in patients with intracanalicular tumors than the middle cranial fossa approach. However, neither differed significantly from the translabyrinthine approach. For hearing preservation, the middle cranial fossa approach was found to be superior to the retrosigmoid approach in patients with tumors <1.5 cm, but not among other size categories. The incidence of CSF leak was significantly greater after the retrosigmoid approach than after either the middle cranial fossa or the translabyrinthine approach. The incidences of residual tumor, mortality, major non-CN complications, residual tumor, tumor recurrence, and dysfunction of other cranial nerves were not significantly different across the three approaches.

Anesthesia dolorosa in these two cases are presented with unilateral facial pain and numbness along the trigeminal nerve. We are speculating the causes of nerve damage due to the iatrogenic (surgical) or radiation induced injury to the trigeminal nerve.

Anesthesia dolorosa is described in the medical literature as the trigeminal nerve distribution that occurs most commonly as a complication of rhizotomy or thermocoagulation to treat trigeminal neuralgia. The prevalence of anesthesia dolorosa is estimated to be in 0 to 1.6% of cases after glycerol rhizotomy [11–14], 0.8 to 2% after radiofrequency rhizotomy [15, 16], and 3% after percutaneous controlled thermocoagulation [17].

The treatment of anesthesia dolorosa typically involves gabapentin [18] and surgery [19]. However, the effectiveness of using gabapentin lacks strong scientific evidence and surgery may be effective in 50% of patients [19].

Anesthesia dolorosa is not a documented complication after acoustic neuroma surgery. The treatment for this complication was thus empirical. In this case series, we report two cases with chronic anesthesia dolorosa after acoustic neuroma surgery. They were treated with multimodality multidisciplinary approaches. The first patient reported satisfactory pain control and with the second patient we were not able to obtain successful pain management.

## 5. Conclusion

This case series provides evidence that anesthesia dolorosa may happen after acoustic neuroma surgery or radiation therapy at or around the trigeminal nerve. It may be difficult to provide enough pain control even with the implementation of thorough multidisciplinary approaches and utilizing pain techniques like nerve blocks and radiofrequency nerve ablation. Anesthesia dolorosa is a centralized pain. Therefore, we may treat these groups of patients the same way we manage thalamic stroke by using deep brain stimulation devices. The electrical neuromodulation of the brain structures (deep brain, periaqueductal gray, and thalamic) may be considered as a treatment modality in the future for these patients.
